# Building Disease Models for Endometriosis: iPSCs as Game-Changers

**DOI:** 10.3390/ijms27125614

**Published:** 2026-06-22

**Authors:** Khalisa H. Kahar, Bushra E-Anjum, Fazlina Nordin, Angela Min Hwei Ng, Nor Haslinda Abd Aziz, Izyan Mohd Idris, Gee Jun Tye, Wan Safwani Wan Kamarul Zaman

**Affiliations:** 1 Department of Tissue Engineering and Regenerative Medicine (DTERM), Faculty of Medicine, Universiti Kebangsaan Malaysia, Jalan Yaacob Latiff, Bandar Tun Razak, Kuala Lumpur 56000, Malaysia; p159646@siswa.ukm.edu.my (K.H.K.); seekbea@gmail.com (B.E.-A.); angela@hctm.ukm.edu.my (A.M.H.N.); 2 Department of Obstetrics and Gynaecology, Faculty of Medicine, Universiti Kebangsaan Malaysia, Jalan Yaacob Latiff, Bandar Tun Razak, Kuala Lumpur 56000, Malaysia; norhaslinda.abdaziz@hctm.ukm.edu.my; 3 Institute for Medical Research (IMR), National Institutes of Health (NIH), Block C, No. 1, Jalan Setia Murni U13/52, Seksyen U13 Setia Alam, Shah Alam 40170, Malaysia; izyan.idris@moh.gov.my; 4 Institute for Research in Molecular Medicine (INFORMM), Universiti Sains Malaysia (USM), Gelugor City 11800, Malaysia; geejun@usm.my; 5 Department of Pharmaceutical Life Sciences, Faculty of Pharmacy, Universiti Malaya, Kuala Lumpur 50603, Malaysia; wansafwani@um.edu.my

**Keywords:** disease modelling, drug screening, endometriosis, induced pluripotent stem cell, patient-derived models, personalized medicine

## Abstract

This review aims to evaluate the potential of endometriosis models, especially patient-derived iPSC models, to gain deeper insights into the disease, thereby advancing our understanding and treatment of endometriosis. This comprehensive narrative review utilized a structured search of the PubMed, Scopus, and Web of Science databases, primarily covering literature published between January 2000 and May 2025. An expansive search strategy was employed to capture the full breadth of the field using keywords such as “endometriosis,” “induced pluripotent stem cells (iPSCs),” “patient-derived organoids,” “disease modeling,” and “epigenetics” without restrictive filtering, ensuring the integration of both foundational theories and emerging biotechnological advances. In total, over 170 peer-reviewed publications were analyzed, ranging from landmark genomic meta-analyses that have identified significant risk loci to state-of-the-art 3D-culture systems for modeling patient-specific endometrial disease. By synthesizing these diverse sources, the review bridges the gap between traditional anatomical classifications and modern molecular modeling to evaluate the potential of iPSC platforms for personalized medicine and therapeutic discovery. Endometriosis is a multifactorial gynecological condition that affects 176 million women worldwide and can significantly impair quality of life. It occurs when endometrium-like tissue grows outside the uterus, responsive to ovarian hormones, causing inflammation, pain, and discomfort, and leading to fibrotic tissue. World Health Organization estimates indicate that 6–10% of women suffer from this disorder, which can cause infertility and increase the risk of developing various types of cancer and autoimmune disorders. The use of patient-derived iPSC models serves to gain deeper insights into the disease by mimicking the endometrial tissue or lesions observed in affected individuals, thereby advancing our understanding and treatment of endometriosis.

## 1. Introduction

Endometriosis is a complex gynecological disorder affecting approximately 6–11% of women of reproductive age worldwide and may exhibit progressive features in a subset of patients, making it a significant global health concern [1,2]. The disease is characterized by the presence of endometrial-like tissue outside the uterine cavity and is frequently associated with chronic pelvic pain, dysmenorrhea, dyspareunia, and infertility, all of which substantially impair physical, psychological, and social well-being. Beyond its clinical manifestations, endometriosis imposes a considerable socio-economic burden on affected individuals and healthcare systems. The annual cost per patient has been estimated at approximately USD 12,419, a financial burden comparable to other chronic diseases such as diabetes, Crohn’s disease, and rheumatoid arthritis [1,3]. Additionally, women with endometriosis reportedly lose an average of 10.8 working hours per week due to absenteeism and reduced workplace productivity (presenteeism), further highlighting the profound impact of the disease on daily functioning and quality of life [1].

Stem cells are extraordinary cells with the remarkable ability to self-renew and differentiate into specialized cell types within the body. They serve as the foundation for all tissue and organ development, vital to growth, repair, and regeneration. Induced pluripotent stem cells (iPSCs) represent a revolutionary advancement in biomedical research, offering a method to generate patient-specific pluripotent stem cells from somatic cells such as skin fibroblasts or blood cells [4]. These iPSCs can differentiate into various cell types of the body, making them invaluable for studying disease mechanisms, drug screening, and potentially regenerative medicine. In the context of endometriosis, iPSCs hold significant promise. Endometrial cells, which can be obtained from the uterus lining, are valuable for generating induced pluripotent stem cells (iPSCs). To date, iPSCs from various sources provide a powerful tool for studying endometrial regeneration [5,6], disease screening [7], and reproductive health [8], as well as for exploring therapeutic applications [9]. These advances show that iPSCs can faithfully recapitulate disease-specific phenotypes in vitro, and therefore the same approach should be feasible for modelling and screening therapeutics in endometriosis as well. To effectively apply this strategy, it is essential to consider the cellular composition and microenvironment of the endometrium, which underpin disease pathogenesis.

The human endometrium comprises two main cell types, epithelial cells, which form the glandular and surface lining, and stromal cells, which provide structural and functional support within the tissue microenvironment. In endometriosis, lesions that develop outside the uterine cavity, such as on the ovaries or peritoneum, are characterized by the presence of both endometrial epithelial and stromal cells, closely mimicking the cellular composition of the standard endometrial lining [10,11,12]. Accurately recapitulating this complex multicellular architecture in vitro is essential for understanding the mechanisms underlying lesion formation and disease progression [13]. Despite this need, traditional modelling approaches remain limited in their ability to mimic such complexity.

**Figure 1:**
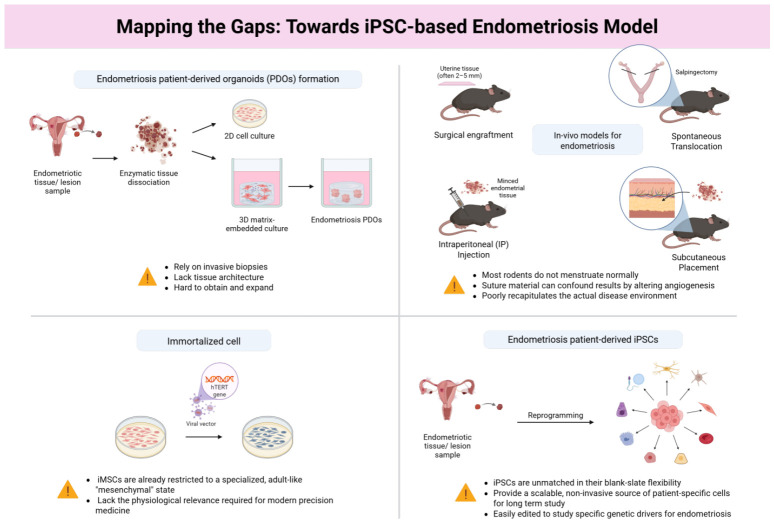
Comparison of existing endometriosis disease models and the emerging role of patient-derived iPSCs. Patient-derived organoids, animal models, and immortalized cell lines each contribute valuable insights into endometriosis biology but are limited by restricted cellular complexity, species-specific differences, or loss of physiological relevance. Patient-derived iPSCs overcome many of these limitations by combining unlimited self-renewal capacity with patient-specific genetic information, enabling the generation of multiple endometrial cell types for disease modeling, mechanistic studies, drug screening, and precision medicine applications. Created in BioRender. Kahar, K. (2026) https://BioRender.com/wxkflai (accessed on 20 April 2026).

Previous studies of two-dimensional (2D) cell cultures lack cellular heterogeneity, do not accurately reproduce extracellular matrix (ECM) organization or cell-ECM interactions, and often show reduced expression of disease-related genes in epithelial and stromal cells [13] (Figure 1). Additionally, 2D systems lack natural diffusion barriers, leading to non-physiological exposure to nutrients, oxygen, and drugs, which limits their utility in faithfully modelling endometriosis [13]. Although primary cell cultures provide high biological relevance, they are frequently constrained by a limited expansion capacity and a tendency to lose their native functional characteristics during prolonged cultivation [13,14]. Immortalized cell lines successfully overcome these longevity issues but often suffer from genetic modifications that may diverge from the original patient physiology [14,15,16]. Clinical biopsies remain a primary source of patient material, yet they are limited by small sample volumes and the ethical complexities associated with repeated invasive surgical procedures [17,18]. Similarly, animal models fail to accurately replicate the unique human hormonal and genetic complexities required to study spontaneous disease progression [14,19,20].

The pioneering development of long-term primary endometrial organoids by research groups such as Boretto et al., Turco et al. [21,22], and more recently Riaz et al. [23] has fundamentally advanced epithelial biology by enabling the robust, sustained expansion of human primary endometrial epithelial cells. While these foundational models provide high-fidelity representations of the endometrial lining and allow for the detailed analysis of hormonal effects, they are predominantly lineage-restricted to epithelial cells and typically lack the integrated stromal and immune components necessary to replicate complex tissue-level crosstalk [6,23,24]. Furthermore, the establishment of these primary cultures remains reliant on initial invasive surgical biopsies, which poses challenges for scalability and longitudinal patient recruitment [14,23]. Consequently, induced pluripotent stem cell (iPSC) technology has emerged as a vital complementary tool that can be integrated alongside these primary organoid systems to create more sophisticated disease models [6,8]. Because iPSCs enable the directed differentiation of both endometrial epithelial and stromal cells from a single, isogenic genetic background, they offer a unique capacity for multilineage modeling that captures the defective stromal-epithelial interactions central to endometriosis [25]. Moreover, iPSC technology provides a solution to the scarcity of primary tissue by offering a virtually limitless and scalable source of patient-specific material that can be generated from entirely non-invasive sources, such as urine-derived cells [26,27,28]. Beyond scalability, iPSCs provide a unique developmental window to study the early ontogeny and initiation of the disease, such as Müllerian duct differentiation, as cells transition from a pluripotent state to a disease-associated lineage, a process largely inaccessible in adult-derived primary tissues [29,30]. Furthermore, contrary to the limitation of other cell-centered platforms, iPSC technology provides a robust framework for investigating immune-mediated defects in endometriosis [5]. iPSCs can be directed to differentiate into functional hematopoietic lineages, including macrophages, dendritic cells, and NK cells, which exhibit morphological and phenotypical characteristics indistinguishable from their in vivo counterparts [31,32,33]. These patient-derived immune cells can be used in functional experiments, such as assessing inflammatory cytokine production and phagocytic capacity, to elucidate how systemic or local immune dysfunction contributes to the survival of ectopic lesions [32,34]. While the complexity of such models has historically been a concern, technological advancements have significantly streamlined these processes; specifically, small-molecule protocols have reduced iPSC induction timelines to as little as 48 h [35], with efficient expansion and cryopreservation achievable within 1–2 weeks [36]. This feasible framework provides a robust platform for investigating immune-mediated defects by differentiating iPSCs into functional hematopoietic lineages, including macrophages, dendritic cells, and NK cells, which exhibit phenotypic characteristics indistinguishable from their in vivo counterparts. The high fidelity of these models is further supported by 2025 evidence demonstrating the successful differentiation of iPSCs into functional decidual natural killer (dNK)-like cells [37] that accurately mirror uterine-specific phenotypes and secretome profiles. These patient-derived immune cells can be used in functional experiments, such as assessing inflammatory cytokine production and phagocytic capacity, to elucidate how systemic or local immune dysfunction contributes to the survival of ectopic lesions [32,34]. By integrating iPSC-derived immune components into endometrial models, researchers can replicate the complex inflammatory crosstalk and immune evasion that define the disease microenvironment [38,39]. When combined with high-throughput CRISPR genetic engineering to interrogate specific risk alleles within standardized, clonally pure populations, this complementary approach becomes essential for elucidating the molecular triggers and reciprocal signaling that facilitate lesion survival and invasion [38,39,40].

Recent advancements in reproductive-lineage differentiation strongly support the feasibility of generating patient-specific iPSC-based models for endometriosis. For example, iPSC-derived Müllerian-duct-like cells (MDLCs) have been produced through stepwise differentiation of human pluripotent stem cells into mesendoderm, intermediate mesoderm, and ultimately MDLCs capable of forming both epithelial and stromal components in 3D culture. When transplanted into a rat model of full-thickness endometrial injury, these MDLCs successfully regenerated functional endometrial glands and restored fertility, demonstrating that iPSC-derived cells can reconstruct complex endometrial architecture and function in vivo [29].

Similarly, patient-specific iPSC lines generated from women with polycystic ovary syndrome (PCOS) have been differentiated into ovarian-lineage cells that accurately recapitulate metabolic, hormonal, and transcriptional abnormalities of the disorder [41,42,43]. These iPSC-PCOS models highlight the ability of iPSC systems to retain patient-specific pathological signatures and serve as powerful “disease-in-a-dish” platforms for studying reproductive dysfunction and testing therapeutic interventions [6,44,45].

Additionally, previous studies [18,19,20] underscore a critical point that iPSCs can faithfully reproduce disease-relevant reproductive-tract phenotypes, maintain patient-specific molecular features, and generate multiple cell types required to model complex tissue interactions. This growing evidence base provides a strong foundation supporting the development of endometriosis patient-derived iPSC platforms as next-generation tools for mechanistic discovery, drug screening, and personalized medicine.

### 1.1. Gaps

Current models of endometriosis remain limited in their ability to capture patient-specific molecular signatures and the dynamic interactions between endometrial cell types. While iPSC technology offers a promising solution to overcome these shortcomings, it shifts the analytical focus toward investigating disease initiation by modeling the patient’s germline susceptibility within the “primed” eutopic-immune interface. Because iPSCs capture the donor’s complete genetic landscape, they can be generated from non-invasive sources such as urine-derived cells or blood, enabling the creation of isogenic models that combine iPSC-derived eutopic endometrial cells and immune cells from the same individual. These systems are uniquely suited to interrogate the endocrine-immune interface, specifically how inherited genetic variants and reduced progesterone responsiveness disrupt the local environment to facilitate the successful survival and implantation of retrograde tissue. Furthermore, the application of CRISPR-based gene editing allows for the refinement of these patient-derived lines into isogenic pairs, isolating the specific impact of risk alleles on the eutopic-immune crosstalk that drives early pathogenesis. Endometriosis disease modelling remains constrained by the incomplete understanding of the underlying pathogenic and epigenetic signatures of endometriosis, which are essential to ensure that iPSC-derived models faithfully recapitulate the disease phenotype. Continued refinement of iPSC-based approaches is therefore critical to advancing more accurate and personalized endometriosis models. This paper provides up-to-date knowledge on endometriosis pathogenesis and clinicopathophysiology and discusses the limitations of current disease models and the feasibility of using patient-derived iPSC models to gain deeper insights into the disease, thereby advancing our understanding and treatment of endometriosis.

### 1.2. The Disease

Endometriosis is a multifactorial medical condition in which tissue similar to the lining inside the uterus, known as the endometrium, starts to grow outside the uterus [46]. This abnormal growth can occur on the ovaries, fallopian tubes, the outer surface of the uterus, and other pelvic organs. In rare cases, endometrial-like tissue can also be found beyond the pelvic region [47]. Symptoms of endometriosis can include dysmenorrhea (painful menstrual cramps), chronic pain in the lower back and pelvis, heavy or irregular periods, fatigue, and infertility. These debilitating symptoms can affect daily activities, work productivity, and overall quality of life [48]. Additionally, endometriosis also poses challenges for women’s reproductive health. It is associated with an increased risk of infertility, as the presence of endometrial tissue outside the uterus can interfere with fertility processes. Many women with endometriosis face difficulties in conceiving and may require specialized reproductive treatments to achieve pregnancy.

**Figure 2:**
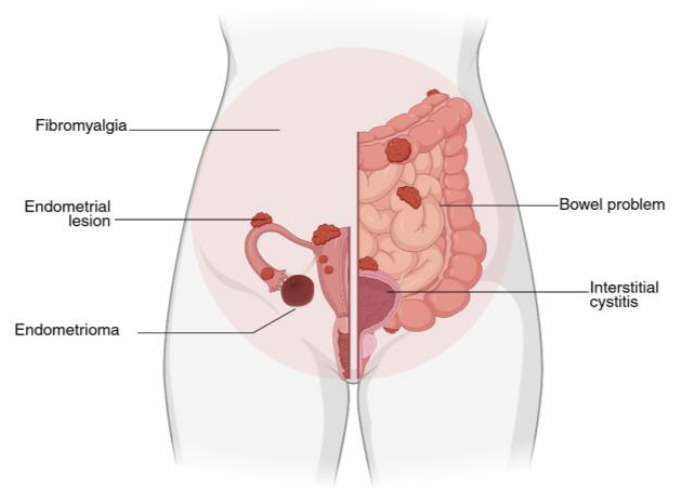
Visualization of endometriotic complications highlighting their distribution and locations within the pelvic cavity. Created in BioRender. Hasyahril, M. (2026) https://BioRender.com/z7x3rci (accessed on 19 August 2025).

Endometriomas (Figure 2) are cysts that form when endometrial tissue grows inside the ovaries. These cysts can cause pain and discomfort, and they may need to be surgically removed [49]. Endometriosis can also affect the bowel and bladder, causing symptoms like painful bowel movements, diarrhea, constipation, painful urination, and occasionally, blood in the urine [50]. In addition to that, endometrial tissue can sometimes grow on the bladder, leading to a condition called interstitial cystitis. This can cause chronic pain and discomfort in the bladder and pelvic area [51]. Additionally, while rare, endometriosis may increase the risk of certain ovarian cancers, particularly endometrioid and clear cell types. Another symptom of endometriosis is fibromyalgia, a chronic pain condition that is often associated with other chronic conditions such as widespread pain and tenderness [52]. While not a primary symptom of the lesions themselves, fibromyalgia often exists as a comorbidity driven by central sensitization state in which the central nervous system becomes hyper-responsive to stimuli [53,54]. In endometriosis patients, the prolonged exposure to peripheral inflammatory signals from lesions (often persisting for the 8–12 years typically required for a diagnosis) can induce permanent changes in pain processing pathways. This leads to cross-organ sensitization, where noxious inputs from the pelvic viscera spread to other regions [54]. These conditions can significantly impact daily activities such as attending school or work, requiring individuals to manage symptoms while maintaining their composure to fulfill their responsibilities. Understanding the etiology and pathology of endometriosis is crucial for improving diagnosis, developing targeted treatments, and enhancing patient outcomes.

The current existing treatments for endometriosis primarily focus on pain management and addressing associated symptoms. Nonsteroidal anti-inflammatory drugs (NSAIDs) are commonly used to relieve pain and reduce inflammation [55]. Hormonal therapies, such as combined oral contraceptives, progestins, and gonadotropin-releasing hormone (GnRH) agonists, can help regulate the menstrual cycle and reduce endometrial tissue growth [56]. Surgical interventions, such as laparoscopic excision or ablation of endometriotic lesions, may be performed to remove or destroy abnormal tissue and alleviate symptoms [57]. In more severe cases or when fertility is a concern, more extensive surgeries like hysterectomy or oophorectomy (removal of ovaries) may be considered.

## 2. Etiology and Pathology

The understanding of the genetic architecture of endometriosis has transitioned from single-locus associations to massive multi-ethnic meta-analyses [58,59]. To date, more than 20 GWASs have been conducted, cumulatively identifying approximately 200 polymorphisms associated with disease risk [60,61]. A landmark 2023 study involving 60,674 cases and 701,926 controls identified 42 genome-wide significant loci and 49 distinct association signals, with the strongest genetic loading observed in stage III/IV disease [61]. These signals implicate genes involved in sex steroid hormone pathways and structural nuclear integrity (see Table 1) [60,62].

The genes identified above in these genomic signals are directly responsible for driving the hallmark biological processes of endometriosis, including estrogen dependence, tissue adhesion, and disease progression. Specifically, genes such as *ESR1* and *FSHB* are central to hormone metabolism and estrogen receptor signaling, providing the molecular basis for the estrogen-dependent growth and survival of ectopic lesions [63,64]. Beyond hormonal drivers, the disease’s “invasive” behavior is linked to structural genes like *FN1*, which encodes fibronectin, a protein that facilitates cell adhesion and migration, essentially acting as the molecular anchor that allows shed endometrial cells to attach to the peritoneal lining [60,64]. Similarly, variants in *SYNE1* affect nuclear envelope integrity, which is believed to play a role in the structural remodeling and cellular motility required for lesions to invade surrounding tissues [60,61]. Finally, certain loci like 7p15.2 (specifically the *rs12700667* variant) serve as clinical indicators of severity, as they are most strongly associated with Stage III and IV endometriosis, where patients experience extensive adhesions and deep infiltrating lesions [61,62]. Collectively, these findings demonstrate that genetic susceptibility contributes not only to disease risk but also to the biological mechanisms underlying lesion establishment and progression. However, the presence of genetic risk variants alone is insufficient to explain why only a subset of individuals develop endometriosis. This has led to the emergence of conceptual frameworks that integrate inherited predisposition with acquired cellular and environmental factors, most notably the “Seed and Soil” model of disease initiation.

### 2.1. The ‘Seed and Soil’ Framework: Germline vs. Somatic Initiation

The pathogenesis of endometriosis is increasingly understood through a “seed and soil” framework that integrates both inherited genetic susceptibility and acquired somatic alterations. Genome-wide association studies (GWASs) have identified numerous germline risk loci associated with endometriosis, including variants at 7p15.2 and other susceptibility regions [62]. Because these variants are germline in origin, they are present in every cell of an affected individual, including both the eutopic endometrium and ectopic lesions [65,66]. Consequently, GWAS findings provide insight into the systemic genetic background, or “soil,” that predisposes certain individuals to disease development by creating a permissive hormonal, immunological, and molecular environment for lesion establishment and persistence [62].

However, inherited susceptibility alone does not fully explain why only a subset of women develop endometriosis despite the high prevalence of retrograde menstruation [67]. Increasing evidence suggests that disease initiation is driven by somatic mutations acquired within the eutopic endometrium before cellular dissemination [68,69]. These mutations effectively act as the “seed,” conferring selective advantages that enable specific endometrial cell populations to survive, evade immune clearance, implant, and proliferate at ectopic sites following retrograde menstruation [65,68,70]. Thus, while germline variants establish a permissive background [62], somatic alterations may represent the critical initiating events that trigger lesion formation.

Recent genomic studies have demonstrated that these initiation-relevant alterations are surprisingly common within histologically normal eutopic endometrium [68,69]. Lac et al. [71] identified somatic driver mutations, including alterations in *KRAS*, *PIK3CA*, and *FGFR2*, in more than 50% of eutopic endometrial samples. Similarly, Inoue et al. [72] reported *KRAS* mutations in 37.1% (26/70) of eutopic endometrial tissues from affected individuals, linking these alterations to disease progression and progesterone resistance. Furthermore, Koppolu et al. [73] detected somatic variants in the eutopic epithelial cells of all patients examined in a cohort with deep infiltrating endometriosis, highlighting the widespread presence of genomic alterations prior to ectopic lesion development. These observations align with broader genomic analyses indicating that extensive genomic heterogeneity and expanded clones of cells carrying significant mutation burdens are a common feature of many normal human tissues, effectively rendering such mutations part of the “new normal” rather than distinct markers of pathology. Consequently, the presence of these genetic alterations alone is insufficient to explain disease initiation or account for the pathogenesis of endometriosis in isolation [71,73]. Instead, these findings underscore the necessity of investigating how such pre-existing genetic architectures interact with the eutopic-immune interface and hormonal dysregulation [68,69]. Given that isolated mutations do not predictably delineate disease status, a more comprehensive approach is required to resolve the complex interplay within the “primed” environment. This highlights the critical utility of patient-derived iPSC models; unlike static genetic analyses, these isogenic platforms preserve the donor’s comprehensive genetic, epigenetic, and immune-regulatory background. This enables the investigation of how specific risk alleles and environmental factors interact with the local endometrial microenvironment to facilitate the retrograde survival and implantation of endometrial tissue.

The importance of the eutopic endometrium as the primary site of disease initiation is further supported by the revised stem cell theory [74], which proposes that the endometrial basalis serves as a reservoir of progenitor or stem cells harboring disease-associated somatic mutations. Upon shedding and dissemination, these genetically altered cells may possess enhanced survival and implantation capabilities, facilitating the establishment of ectopic lesions [68,74]. This concept provides a mechanistic link between the eutopic endometrium and the development of endometriosis at distant sites.

In addition to genetic sequence variations, epigenetic modifications are now recognized as primary drivers of the endometriosis phenotype. Recent investigations have identified 51 methylation quantitative trait loci (mQTLs) distributed across 21 genomic loci that influence disease susceptibility by modulating the expression of DNA methyltransferases and demethylases [75,76]. These enzymatic shifts lead to “epigenetic scars” and altered chromatin architecture, particularly affecting the steroid hormone signaling pathways that define endometrial cell identity [76,77]. Complementing this, micro-RNAs (miRNAs) provide an additional layer of post-transcriptional control; for instance, miR-202-3p, miR-424-5p, and miR-556-3p have been identified as key contributors to the abnormal angiogenic activity observed in ovarian endometrioma, while miR-449b-3p and miR-29c-3p are associated with the impaired tissue-remodeling capabilities of deep infiltrating lesions [78]. These dysregulated miRNA profiles are further linked to the hallmark progesterone resistance of the disease, hindering the normal decidualization process [75]. Crucially, these molecular signatures are being translated into non-invasive clinical tools; a validated 109-miRNA saliva signature recently demonstrated an accuracy of 96% in diagnosing superficial peritoneal endometriosis, markedly outperforming conventional imaging [79,80]. Collectively, these epigenetic aberrations offer a critical molecular framework for developing high-fidelity, patient-specific iPSC models that can replicate the complex, individualized pathogenesis of the disease [77].

### 2.2. Key Hypotheses Describing the Origin and Progression of Endometriosis

While these genetic and epigenetic insights explain the molecular “how” of lesion survival, they are fundamentally tied to the long-standing theories regarding the “where” and “why” of the disease’s origin. The most prominent of these, the transplantation and dissemination theory (Table 2) integrating the frameworks proposed by Sampson and Halban, suggests that the disease arises from the transport and subsequent implantation of viable endometrial tissue into ectopic sites [81,82]. This hypothesis builds upon the phenomenon of retrograde menstruation with the reflux of menstrual debris through the fallopian tubes, by proposing that viable endometrial cells within this reflux possess the unique capacity to adhere to, invade, and proliferate upon the peritoneal surface [67,69]. Although some studies report its occurrence in up to 90% of women [67], clinical observations by Vercellini and colleagues suggest more conservative estimates of approximately 50–60%, depending on the timing and method of observation [83].

Importantly, retrograde menstruation itself should be distinguished from Sampson’s theory; while retrograde menstruation describes the physical dissemination of endometrial cells into the peritoneal cavity, Sampson’s implantation/transplantation theory further proposes that specific molecular, cellular, and immune-related abnormalities enable these displaced cells to evade clearance, survive, implant, and establish ectopic lesions [84,85].

This is where the previously identified risk factors such as *FN1* variants for adhesion and *ESR1* variants for estrogen sensitivity provide the missing link, explaining why only certain individuals possess the cellular environment that allows misplaced tissue to survive, adhere, and invade [60,64]. To address these limitations and explain the presence of distant, extrapelvic lesions, the theory incorporates lymphatic and vascular dissemination, which describes the systemic spread of endometrial cells through the circulatory and lymphatic systems [82]. In addition, the coelomic metaplasia theory suggests that multipotent peritoneal cells can transform into endometrial-like tissue, accounting for rare cases such as endometriosis in men or at distant sites (e.g., deep infiltrating endometriosis, adenomyosis) [86]. While this concept has historically addressed the emergence of endometriosis in extrapelvic sites, it is increasingly criticized in modern literature [87,88]. Contemporary analyses, such as the review by J Clin Med, highlight significant challenges to this model; notably, conclusive experimental proof for such transformations remains elusive [88,89,90]. Furthermore, unlike metaplasia observed in other organ systems, endometriosis does not demonstrate the expected age-dependent increase in incidence, presenting a significant hurdle to the validity of this theory as a primary driver [88].

Complementing existing models, the Stem/Progenitor Cell Theory has been proposed to account for lesion formation and disease persistence [84,91,92]. However, this hypothesis faces significant conceptual and empirical challenges that undermine its role as a primary pathogenic explanation. First, the theory often relies on the premise that stem cells are derived from the basalis layer of the endometrium, yet physiological evidence confirms that the basalis is not typically shed during menstruation, representing a critical inconsistency in models suggesting its transplantation [91,93,94]. Second, it remains highly improbable that epithelial and stromal stem cells could migrate and uniformly establish lesions across diverse, non-contiguous anatomical sites, such as the bladder, peritoneum, and ligaments [87,93,95,96].

Furthermore, the characterization of uterine epithelial stem cells remains debatable. The reliance by certain research groups on N-cadherin, a protein classically recognized as a mesenchymal marker, to identify epithelial progenitors is increasingly viewed as problematic and insufficient for accurate cell classification [74,91,94]. Finally, empirical evidence for this theory is lacking, as progenitor cells are only rarely detected within established lesions. Quantitative studies estimate these cells constitute only approximately 0.1% of stromal cells and 0.04% of epithelial cells within endometriotic tissue, often exhibiting limited clonogenic capacity compared to healthy tissue [91,97,98]. Although these cells constitute only a minute fraction of the total cell population, their presence may explain the clonal nature of endometriosis, where identical somatic mutations are often shared across different lesions in the same patient [68,99].

Similarly, the Müllerian remnants theory proposes that embryological residues may give rise to endometriotic lesions in unusual or distant organs [100]. Currently, the Müllerian remnant theory remains largely hypothetical and speculative, as proving the persistence of these rests from embryogenesis to adulthood is methodologically challenging [88]. The Müllerian remnant theory is supported by clinical observations where retrograde menstruation is biologically impossible, most notably in patients with Mayer–Rokitansky–Küster–Hauser syndrome who develop endometriosis despite lacking a functional uterus and menstruation [101,102]. This empirical evidence is further strengthened by rare cases of male endometriosis involving the prostatic utricle, a known vestigial Müllerian remnant [88,89], as well as the identification of ectopic endometrial-like tissue in human fetuses prior to birth [88,103]. Additionally, the theory accounts for the diagnosis of the disease in pre-menarcheal girls, providing a framework for pathogenesis when menstrual reflux cannot occur [87,88].

Regarding disease recurrence, Sampson’s theory remains a more robust explanation for pelvic disease, as it provides a clear anatomical path for the retrograde menstruation of endometrial tissue [92,96]. Current evidence suggests that focusing on systemic populations, such as bone marrow-derived mesenchymal stem cells, is less robust than analyzing persistent dysfunction within the eutopic endometrium and local immune environment [69]. Recurrence is more effectively explained by the underlying endometrial and immune pathology that remains untreated after surgical excision [104]. This conclusion is further supported by the high clinical efficacy of hormonal suppression therapies, such as oral contraceptives, which manage symptoms and restrict disease progression by suppressing the pathological endometrium itself [69]. As a result, recurrence is more accurately understood as the ongoing manifestation of persistent endometrial dysfunction rather than the result of circulating stem cell populations.

Finally, the immune dysfunction theory might provide insight into why only some women with retrograde menstruation develop endometriosis, linking impaired immune surveillance and chronic inflammation to disease persistence and progression [85,105,106].

**Table 2:** Summary of the Key Hypotheses Describing the Origin and Progression of Endometriosis.

Theory	Key Points
The Transplantation and Dissemination Theory	Endometrial cells flow backward through the fallopian tubes into the peritoneal cavity during menstruation, implanting and forming lesions [81]. Viable cells further disseminate to distant anatomical sites through the lymphatic and hematogenous systems [82].
Coelomic metaplasia theory	Peritoneal mesothelial cells transform (metaplasia) into endometrial-like cells under certain stimuli [86]. Modern critiques note the lack of age-related incidence patterns and the absence of conclusive experimental proof [90].
Müllerian (Meyer’s) remnants theory	Embryonic Müllerian duct remnants in ectopic locations differentiate into endometrial tissue later in life [100].
Stem/progenitor cell theory	Circulating bone marrow-derived or endometrial stem cells migrate and differentiate into ectopic endometrial tissue [88].
Immune dysfunction theory	Altered immune surveillance or peritoneal immune environment allows ectopic implantation and survival of endometrial cells [85].
Induction theory	This theory suggests that endometriosis can be caused by certain environmental factors or chemicals that induce the growth of endometrial tissue outside the uterus [107].

### 2.3. Endometriosis Heterogeneity

Each patient’s experience with endometriosis is unique, and tailoring treatments to individual needs is critical for improving outcomes. While there is still much research to be done, investigations have already begun to comprehend disease mechanisms and effectively recapitulate them in these models [108,109]. This fundamental work is crucial not only for unraveling how endometriosis behaves at a cellular and molecular level but also for developing more accurate models that faithfully recapitulate the disease. Ultimately, the models will help deepen our understanding of disease mechanisms and lay the foundation for future personalized therapeutic approaches.

**Figure 3:**
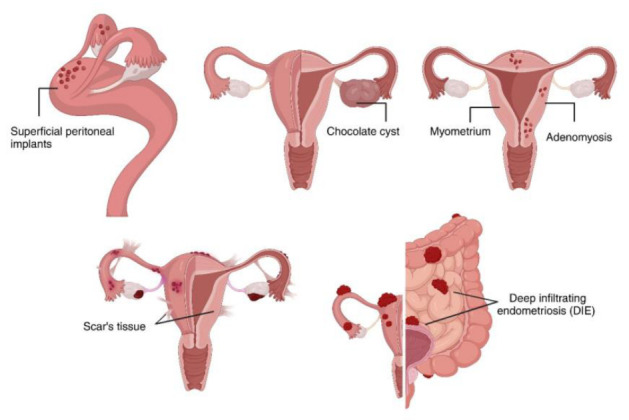
The hallmark of endometriosis is the presence of endometrial glands and stroma in ectopic locations. These lesions can exhibit different morphological patterns, including superficial peritoneal implants, ovarian endometrioma, deep infiltrating endometriosis (DE), and adenomyosis. Each subtype has distinct histological features and clinical implications. Created in BioRender. Hasyahril, M. (2026) https://BioRender.com/z6osxz6 (accessed on 19 December 2024).

The morphology of endometriotic lesions provides important insights into the pathogenesis and progression of the disease. Recent studies have shed light on the various morphological aspects of endometriosis. Figure 3 illustrates the different types and histological features of endometriotic lesions. Superficial peritoneal implants are characterized by the presence of endometrial glands and stroma on peritoneal surfaces [110]. This is depicted in Figure 3, where these implants are shown as red, black, or white lesions with varying degrees of inflammation and fibrosis. The ovarian endometrioma, also known as chocolate cysts, are cystic ovarian masses filled with altered menstrual blood and surrounded by a fibrous capsule [111]. They are different from the typical ovarian cysts which are fluid-filled sacs that form on or inside the ovaries and arise for various reasons, including normal ovarian function (functional cysts) or other benign growths [112]. On the other hand, patients who suffer from deep infiltrating endometriosis (DE) experience significant pain during their menstrual cycle due to the lesions that cause adhesions and sometimes organ dysfunction. DE is characterized by the invasion of endometriotic tissue into the surrounding structures, such as the uterosacral ligaments, rectovaginal septum, and bladder [111]. Histologically, DE lesions show glandular and stromal components within the affected tissues. These pathological processes can promote the development of adhesions and extensive tissue remodeling within the pelvic cavity, which may further aggravate pain, infertility, and treatment complexity. While these manifestations were traditionally described as “scarring,” growing evidence indicates that fibrosis represents a more accurate and central pathological feature of endometriosis. Fibrosis is increasingly recognized across all major forms of the disease, including peritoneal, ovarian, and deep infiltrating endometriosis [113,114]. It is characterized by excessive extracellular matrix deposition and collagen accumulation resulting from the transdifferentiation of fibroblasts into activated myofibroblasts [113,115]. This fibrotic remodeling contributes substantially to tissue stiffness, adhesion formation, pelvic pain, and organ dysfunction. Notably, fibrotic lesions may persist even in the absence of detectable epithelial or stromal endometriotic components, suggesting that fibrosis is not merely a secondary repair response but a fundamental hallmark of disease persistence. Consequently, endometriosis is increasingly regarded as a fibrotic condition rather than solely an ectopic inflammatory disorder [114,116]. While these ectopic forms occur outside the uterine cavity, adenomyosis represents a distinct phenotype where endometrial glands and stroma infiltrate the uterine myometrium, causing uterine enlargement and significant reproductive dysfunction [112,117]. Despite their different anatomical locations, all three phenotypes are increasingly viewed through the lens of a shared pathogenic foundation, where disrupted cellular mechanisms and fibrotic remodeling represent the fundamental hallmarks of disease progression and persistence [114,116].

One of the central challenges in endometriosis research is to precisely understand how patient-specific epigenetic changes originate and drive disease progression, which remains a critical barrier to developing fully representative in vitro models and effective therapeutic strategies. Each individual inherits a unique combination of genetic and epigenetic modifications from their parents at conception, and additional changes can arise during fetal development. After birth, further genetic and epigenetic alterations continue to accumulate over time, driven by factors such as errors during cell division, exposure to environmental toxins, oxidative stress, and radiation.

For example, retrograde menstruation is a common phenomenon in many women, yet only a subset goes on to develop endometriosis [118]. This suggests that individual differences in epigenetic regulation, along with other genetic, hormonal, and immune factors, likely determine susceptibility. These underlying variations help explain why “one-size-fits-all” treatments often fail, as they do not address the complex, patient-specific mechanisms driving disease development. Another study on the molecular level and the metastasis of endometriosis lesion found that different subtypes of endometriosis (ovarian, deep infiltrating, and superficial) often share identical somatic cancer-driver mutations within the same patient, indicating clonality across lesions regardless of anatomical type. Ovarian endometrioma showed higher mutational complexity, frequently containing multiple functionally redundant mutations, suggesting the presence of several clones within a lesion. These results imply that endometriosis can spread as groups of related clones and that current anatomical classifications do not fully capture the disease’s molecular diversity, highlighting the need for classification systems that include genomic features [119,120]. These findings highlight an important challenge for creating faithful disease models of endometriosis, whereby the clonal relationship and potential dissemination of lesions suggest that endometriosis is not a uniform disease even within a single individual. Thus, models must account for molecular and genomic heterogeneity rather than relying solely on anatomical classifications. Incorporating these insights is crucial for developing representative in vitro systems that can more accurately reflect disease complexity and guide personalized therapeutic strategies.

While the creation of fully faithful iPSC-based disease models requires a comprehensive understanding of the genetic and epigenetic landscape of endometriosis, these models also serve as powerful exploratory tools. By reprogramming patient-derived cells and differentiating them into relevant endometrial lineages, researchers can begin to uncover disease-specific molecular and cellular mechanisms. In this way, iPSC-derived models both rely on and contribute to advancing our understanding of endometriosis, creating a dynamic, iterative process of model refinement and biological discovery. Thus, iPSC models are not merely final endpoints but serve as crucial tools to dissect disease pathogenesis, with the potential to evolve into highly accurate disease replicas as knowledge progresses.

## 3. Existing Models

In vitro disease models are essential tools for unraveling disease mechanisms, screening potential therapeutics, and identifying molecular targets; however, as mentioned earlier, traditional 2D cultures and animal models have significant limitations. Two-dimensional monolayer cultures fail to replicate the three-dimensional architecture, cell–cell interactions, and microenvironment found in vivo, resulting in altered gene expression and cellular behavior, and they cannot accurately represent key disease processes such as epithelial–mesenchymal transition, hypoxia, angiogenesis, and inflammation [121].

According to Greaves et al. [122], they face inherent challenges due to anatomical and physiological species differences. As noted by Burns et al. [123], the mouse’s status as a non-menstrual species with a closed reproductive system requires artificial induction methods that may confound the study of spontaneous disease initiation and early cellular attachment [122,124]. Furthermore, the reliance on inbred strains and the fact that human lesions are not yet fully subtyped makes it difficult to model the diverse genetic predispositions and dynamic, stage-specific changes seen in patients [125].

Building upon these established foundations, three-dimensional (3D) in vitro models, particularly those utilizing patient-derived iPSCs, provide a critical bridge toward higher translational fidelity [123]. By transitioning from 2D systems to 3D organoids, researchers can better mimic the human-specific in vivo microenvironment and investigate molecular pathways within a patient-specific genetic context. This advanced approach enables more predictive drug testing [121] and the study of early human-specific pathogenesis without the ‘immunocompetence paradox’ often encountered when human tissue is placed in immunocompromised mice [123,126,127,128]. Ultimately, developing these high-fidelity 3D human models is essential for overcoming traditional species-specific limitations and advancing toward personalized therapeutic strategies [129].

Table 3 was constructed to highlight key findings from existing studies on endometrial-derived models, highlighting both their demonstrated capabilities and their potential future applications. Advances in endometrial organoid technology have demonstrated substantial progress in recapitulating both physiological and disease-specific features of the endometrium, although several limitations remain. Early foundational studies by Turco et al. and Boretto et al. [21,22] established the first genetically stable and ovarian hormone-responsive endometrial organoids derived from glandular epithelium, laying the groundwork for subsequent developments. Building on this, Francés-Herrero et al. [130] demonstrated that these organoids faithfully recapitulate key in vivo glandular characteristics, including gland-like architecture and functional epithelial activity such as glycogen production and MUC-1 secretion into the luminal compartment. Importantly, these models also exhibit chromosomal stability over extended culture periods and can be expanded under optimized conditions, such as with tissue-specific matrices like EndoECM, highlighting their utility for long-term studies.

More recent work has expanded the applicability of organoids to disease modeling. Liu et al. [131] successfully generated patient-derived organoids (PDOs) across major molecular subtypes of endometrial cancer, preserving key genetic features such as mismatch repair status, p53 alterations, and POLE mutations, while Bednarek et al. further confirmed strong concordance between PDOs and primary tumors in both morphology and genomic alterations. Similarly, earlier work by Boretto et al. [133] demonstrated that organoids derived from endometriosis and endometrial cancer patients capture clinically relevant heterogeneity. Together, these findings underscore the strength of organoid systems in maintaining patient-specific traits; however, variability in establishment success rates (e.g., ~71% reported by Liu et al. [131]) suggests that technical and biological constraints still limit universal applicability.

Functionally, these models have proven valuable for drug screening and pathway interrogation. Liu et al. [131] highlighted that 3D PDOs exhibit drug response profiles distinct from matched 2D cultures, emphasizing their superior physiological relevance for preclinical testing. This is further supported by Boretto et al. [133], who demonstrated their suitability for high-throughput drug screening, and by Bednarek et al. [132], who showed that targeted therapies such as T-Dxd, particularly in combination with MEK inhibitors, significantly reduce growth in human epidermal growth factor receptor 2 (HER2)-positive organoids while modulating signaling pathways (e.g., decreased HER2 and increased pERK1/2). These findings highlight the potential of organoids for therapeutic stratification, although the absence of systemic factors (e.g., immune components and vascularization) remains a limitation when translating results in vivo.

Mechanistically, organoids also provide a platform to investigate disease initiation and progression. Francés-Herrero et al. [130] emphasized the role of extracellular matrix remodeling in early endometriosis pathogenesis, enabling the study of how matrix metalloproteinases influence ectopic cell survival and adhesion. Complementing this, Liu et al. [131] utilized ascites-derived organoids to model advanced disease stages and metastatic behavior. Furthermore, rigorous validation approaches, including immunophenotyping (e.g., pan-cytokeratin positivity and absence of vimentin) and stemness marker expression, confirm the epithelial identity and progenitor characteristics of these models, reinforcing their biological relevance.

Overall, while endometrial organoids represent a powerful and increasingly sophisticated platform that bridges basic biology and translational research, challenges such as incomplete microenvironmental representation, variability in establishment efficiency, and limited modeling of systemic interactions must be addressed. Integrating organoids with complementary systems, such as stromal co-cultures, immune components, and microfluidic platforms, will be essential to fully realize their potential in precision medicine and disease modeling.

## 4. Patient-Derived iPSC as Disease Model for Personalized Treatment

Despite their individual limitations, existing non-iPSC disease models mentioned above have collectively demonstrated that key disease-associated features, including aberrant hormone responsiveness, inflammatory signaling, altered cellular differentiation, and pathological tissue behavior, can be faithfully recapitulated ex vivo. The success of these platforms provides critical proof-of-concept that endometriosis-related phenotypes are, at least in part, intrinsically encoded within patient-derived cells rather than being solely imposed by the microenvironment. This concept is reinforced by a growing body of evidence from iPSC-based disease models across reproductive and imprinting-related conditions (Table 4), where patient-specific pathological traits are preserved following reprogramming. For example, iPSC models of trisomy 21 [134] and NLRP7-associated complete hydatidiform mole have successfully recapitulated trophoblast differentiation defects and hyperplastic behaviors observed in primary patient tissues [135], while iPSC-derived trophoblasts from early-onset preeclampsia cases exhibit abnormal stress and oxygen-response phenotypes [136,137]. Similarly, imprinting disorders such as Angelman, Prader–Willi, and Beckwith–Wiedemann syndromes [138,139,140] demonstrate retention of disease-specific epigenetic and transcriptional abnormalities, providing compelling evidence that heritable disease features can survive pluripotent reprogramming.

Within this context, patient-derived induced pluripotent stem cell (iPSC) models emerge as a logical and biologically grounded extension of existing endometriosis modelling strategies. The ability of non-iPSC systems to capture disease phenotypes strongly supports the expectation that these features can also be retained within iPSC-derived lineages that preserve the patient’s genetic background. Indeed, endometrium-derived iPSCs (hEm-iPSCs) established from discarded surgical tissue have demonstrated robust differentiation capacity [5], while hiPSC-derived mesenchymal stem cells have been successfully applied in three-dimensional scaffold-based uterine repair models [141], highlighting the scalability and regenerative potential of this platform. Furthermore, directed differentiation of iPSCs toward Müllerian duct, tubal epithelium, and primordial germ cell lineages [142,143,144] provides unprecedented access to otherwise inaccessible developmental stages and tissue types that are highly relevant to gynecological disease modelling.

Importantly, iPSC-based models should not be viewed as replacements for established primary or organoid systems, but rather as complementary tools that build upon the biological validity already demonstrated by non-iPSC platforms while enabling deeper mechanistic interrogation. By integrating the patient specificity validated in primary and organoid cultures with the experimental control, lineage flexibility, and scalability unique to iPSCs, this approach offers a powerful strategy to overcome persistent limitations in endometriosis research. Collectively, these advances position iPSC-based models as a critical component of next-generation disease modelling frameworks, with strong potential to enhance mechanistic insight, improve disease stratification, and accelerate the development of predictive, personalized, and translationally relevant therapeutic strategies.

**Table 4:** Patient-derived iPSC models for modeling gynecological and developmental diseases.

Model Type	Description of iPSC Model	Refs.
Trisomy 21 (T21)	First genetic model used to validate iPSCs for placental disease; iPSC-derived trophoblasts successfully recapitulated the differentiation defects seen in primary patient cells.	[134]
NLRP7 Mutations	Derived from maternal skin biopsies to model familial complete hydatidiform mole (CHM); demonstrated expedited trophoblast differentiation similar to patient tissue hyperplasia.	[135]
Early-onset Preeclampsia (PE)	Derived from umbilical cord cells; trophoblasts differentiated from these iPSCs showed abnormal responses to oxygen tension, hinting at underlying susceptibility to stressors.	[136,137]
Endometrium-derived iPSCs (hEm-iPSCs)	Established from discarded endometrial tissue post-hysterectomy; successfully used for directed differentiation into hematopoietic and erythroid (red blood cell) lineages.	[5]
hiPSC-derived MSCs (hiMSCs)	Human iPSC-derived Mesenchymal Stem Cells loaded onto 3D-bioprinted hydrogel scaffolds; used for promoting recovery and repair of the uterine endometrium in injury models.	[141]
Müllerian Duct/Tubal Origin	iPSCs directed to differentiate into intermediate mesoderm of tubal origin, which can further develop into Müllerian ducts and the female reproductive tract epithelia.	[142,144]
Primordial Germ Cell Lineage	Human iPSCs cultured for 1–2 weeks with specific growth factors (BMP-4, 7, 8b) to differentiate into the earliest lineage of the human germ line.	[143]
Angelman Syndrome	Reprogrammed from patients with this imprinting disorder; proved that aberrant DNA methylation patterns present in the patient’s somatic cells are retained in the iPSCs.	[139]
Prader–Willi Syndrome	Patient-derived models that successfully recapitulate the expected transcriptional and epigenetic features associated with the disease pathogenesis.	[138]
Beckwith–Wiedemann Syndrome	Represents the first human cell-based model of this syndrome, established through iPSC technology to study parent-of-origin imprinting abnormalities.	[140]

### 4.1. Directed Differentiation of iPSCs into Functional Endometrial Lineages

The primary breakthrough in differentiating human induced pluripotent stem cells (iPSCs) into endometrial lineages was achieved by Miyazaki et al. [144], who developed a highly defined 14-day differentiation protocol that mimics the embryonic development of the female reproductive tract [29,30,109]. This method utilizes a 3D embryoid-body-based approach to guide iPSCs through successive developmental milestones, beginning with their transition into the intermediate mesoderm (IM) by day four [30,109]. These IM-like cells are characterized by the expression of key urogenital markers, such as LHX1 and PAX2 [144]. The protocol’s developmental authenticity is further highlighted by the fact that these IM cells are bi-potential; while they can be directed toward a renal identity, BMP inhibition prevents this path, while WNT activation successfully directs them toward the Müllerian duct (MD) stage by day eight [29,30,144].

Following the establishment of MD-like cells, the final stage of the protocol involves a specific signaling cocktail to generate functional endometrial stromal fibroblasts (EMSFs) [30,144]. For an additional six days, the cells are treated with 5′-aza-2′-deoxycytidine (5aza2), CHIR99021, 17β-estradiol (E2), FGF9, and PDGF-BB. The resulting iPSC-derived EMSFs demonstrate a molecular signature nearly identical to native endometrial stroma, expressing critical markers such as *HOXA10, HOXA11*, and *PGF*. RNA sequencing has further confirmed that these cells share a highly similar transcriptional profile with primary endometrial stromal cells isolated from patients [30,144].

The most significant achievement of this model is its functional hormone responsiveness, specifically the ability of iPSC-derived EMSFs to undergo decidualization [144]. When treated with a cocktail of E2, progestin, and cyclic adenosine monophosphate (cAMP) for eight days, the cells transform into a secretory state essential for embryo implantation. This process is validated by the significant up-regulation of decidualization markers, including *FOXO1*, *HAND2*, *IGFBP1*, and *PRL* [144]. These achievements provide a scalable and genetically flexible platform for “disease-in-a-dish” modeling of conditions like endometriosis and endometrial cancer, as well as for high-throughput drug screening and potential future applications in regenerative medicine and uterine reconstruction [22,30,109].

### 4.2. Cell Sources

While primary endometriotic cells derived from lesions offer high authenticity of the disease’s end-state [13], it is increasingly recognized that the eutopic (uterine) endometrium is the actual driver of disease initiation and recurrence [69]. Recent molecular evidence demonstrates a clonal continuity between uterine endometrial epithelium and ectopic lesions, suggesting that the ‘source’ tissue in affected women already possesses the cancer-associated mutations and epigenetic defects required for survival outside the uterine cavity [68,99]. Therefore, focusing solely on ectopic lesions may overlook the early-stage mechanisms that prime these cells for invasion and persistence. Consequently, the ideal endometriosis model must bridge the gap between the source (eutopic) and the result (ectopic). iPSC platforms are uniquely positioned to do this by deriving iPSCs from the uterine endometrium of patients, and researchers can model the transition of these ‘primed’ cells into an aggressive phenotype in a controlled 3D environment [13]. This approach allows for the investigation of systemic defects in the patient’s underlying genetic and epigenetic landscape, which are the true drivers of the disease’s lifecycle, from initial shedding via retrograde menstruation to successful implantation and subsequent recurrence [69,145].

Additionally, induced pluripotent stem cells (iPSCs) present a patient-specific and expandable alternative. Generated iPSCs from a patient’s somatic cells, including endometrial cells or even less invasive sources like urine-derived cells [26], which can be expanded indefinitely, offer a virtually unlimited supply for research and potential therapies. Theoretically, iPSCs can differentiate into any cell type, including endometrial stromal fibroblasts, allowing researchers to model disease features within a patient’s genetic background [144]. Despite these advantages, iPSCs have notable limitations; they may acquire new mutations during reprogramming and expansion, introducing genetic drift that can affect function and reproducibility. Furthermore, differentiation protocols may not fully produce mature, disease-relevant cell types, resulting in models that only partially reflect the true disease phenotype [146]. Variability between iPSC lines, even from the same patient, can also complicate reproducibility and consistency across studies [147]. A key uncertainty in iPSC-based endometriosis modeling concerns the fidelity of epigenetic mark preservation. Extensive epigenetic remodeling occurs during reprogramming to pluripotency.

However, successful precedents from epigenetically driven diseases suggest otherwise. Examples from imprinting-related disorders, where epigenetic modifications are central to disease, indicate that disease-specific epigenetic signatures can persist through reprogramming. Prader–Willi, Angelman, and Beckwith–Wiedemann syndrome iPSC models all retain characteristic epigenetic abnormalities, suggesting that endometriosis-derived iPSCs may similarly preserve disease-relevant epigenetic features if they are functionally important to the disease. This precedent justifies empirical investigation of epigenetic mark fidelity in endometriosis models.

Therefore, obtaining tissue from the disease-specific site is a critical consideration for developing biologically relevant endometriosis models, as the local microenvironment plays a central role in shaping disease-associated cellular behavior. Endometriotic lesions are characterized by tissue-resident immune cell-driven inflammatory responses, aberrant extracellular matrix remodeling, and sustained exposure to pro-inflammatory cytokines, all of which contribute to the survival, adhesion, and proliferation of ectopic endometrial cells [120]. In addition, dysregulation of developmental pathways, including Homeobox genes and Wnt/β-catenin signaling during Müllerian duct development, has been implicated in aberrant cell placement and mesodermal differentiation [89]. Together, these findings support the rationale that sourcing cells from disease-affected sites may increase the likelihood of capturing molecular and epigenetic features shaped by the pathological microenvironment. When applied to patient-derived iPSC modelling, disease-site cell sourcing may therefore provide a biologically informed foundation for investigating disease mechanisms and improving the relevance of personalized in vitro endometriosis models.

Previous studies in other disease contexts have demonstrated that sourcing cells directly from the affected tissue preserves unique molecular and epigenetic signatures, enabling more precise investigation of disease mechanisms and progression [148,149]. Applying this principle to endometriosis would increase the likelihood of capturing the authentic cellular behavior and pathological features of the disease in vitro.

## 5. Shortcoming and “Building Block Strategy”

**Figure 4:**
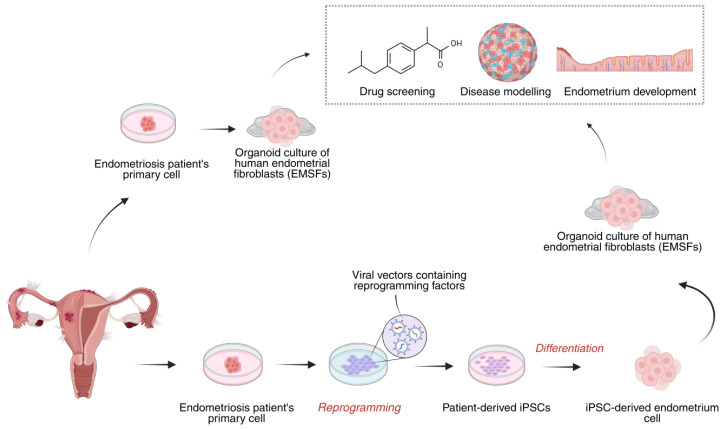
Potential application of induced pluripotent stem cells (iPSCs) in personalized treatment approaches for individuals with endometriosis. Created in BioRender. Hasyahril, M. (2026) https://BioRender.com/8yh8cqu (accessed on 22 April 2025).

Utilizing iPSCs to create these organoids is particularly advantageous because it allows for the derivation of patient-specific cells directly from tissues affected by endometriosis, enabling personalized medicine approaches (Figure 4). As mentioned, endometriosis is a highly heterogeneous disease, with molecular profiles and clinical symptoms varying significantly between individuals. The development of induced pluripotent stem cell (iPSC)-based “living biobanks” offers a powerful approach to capture the unique genetic and epigenetic signatures of diverse patients. This platform enables precision medicine strategies, allowing for personalized drug screening to identify therapies that are effective for specific patient subtypes, thereby addressing the limitations of the current one-size-fits-all treatment paradigm. Building reliable models of endometriosis remains a considerable challenge. Difficulties such as obtaining pure and representative samples from the disease site, ensuring accurate characterization, working with a sufficient number of samples, and the technical skills required all add complexity to the process. These challenges are further compounded by the heterogeneity of lesions, differences in patient backgrounds, and the fact that the pathogenesis of endometriosis is still not fully understood. If these barriers can be addressed, however, more accurate models could be developed, opening the door to a wider range of therapeutic approaches, one of which is the use of endometriosis-derived iPSCs for cancer biomarker screening [150].

While induced pluripotent stem cells (iPSCs) offer a promising solution, their application is not without limitations. iPSCs may retain epigenetic memory from their donor cell type, potentially restricting their differentiation into unrelated lineages, and they also carry a risk of tumorigenicity, particularly the formation of teratomas if differentiation is incomplete or inaccurate. 

To address these limitations, a “building block” strategy has been proposed, where iPSCs are not used in isolation but instead serve as a source of epithelial and stromal cells for integration into advanced three-dimensional (3D) models such as assembloids [6,130]. This synergistic approach enhances physiological relevance and enables the study of complex interactions, including the endometrial–peritoneal interface (EPI), in a genetically precise and patient-specific manner [151]. For researchers and clinicians, this integrated approach facilitates time-resolved interrogation of early disease initiation, a stage that remains largely inaccessible due to the delayed clinical presentation of the disease. These models enable the investigation of specific epigenetic “switches”, such as the promoter hypermethylation of decidualization genes like HOXA10 which may prime uterine cells for an aggressive phenotype before they even reach the peritoneal cavity [6,109,144]. Ultimately, the creation of these patient-specific biobanks provides a vital bridge between understanding fundamental disease mechanisms and advancing precision medicine and early intervention strategies for individuals with endometriosis.

If these barriers can be overcome, more robust and representative models could be developed, opening new avenues for therapeutic discovery. One such application is the use of endometriosis-derived iPSCs for cancer biomarker screening. Unlike blood-based screening, which may miss important signals, lesion-derived iPSCs capture disease-specific molecular changes directly from the affected site [152,153]. This is especially important given the well-documented link between endometriosis and endometriosis-associated ovarian cancer such as ovarian clear cell carcinoma, where identifying early molecular alterations could help determine whether a patient is at greater risk [154]. By generating patient-specific iPSCs and reconstructing disease-relevant cellular environments in vitro, researchers can investigate early malignant transformation, identify predictive biomarkers, and assess individual cancer susceptibility with greater precision.

Ultimately, this strategy could support the establishment of living biobanks, advance personalized treatment approaches, and improve early detection and intervention. In this way, endometriosis-derived iPSCs serve as a critical bridge between understanding disease mechanisms and enabling precision medicine in endometriosis-associated cancers. Overall, iPSC technology represents a transformative tool in advancing our understanding and treatment of endometriosis, bridging the gap between basic research and clinical application. In the context of disease modeling, understanding its pathology is crucial for accurately mimicking the disease conditions in the iPSCs-derived model, which includes cellular abnormalities, inflammatory responses, tissue remodeling and potential complications.

## 6. Reprogramming of Cells into iPSC and Its Challenges

Through the years, many methods were established to realize the reprogramming of desired cells into iPSC. This is to ease the delivery of vectors containing desired transcription factors, as well as to reduce safety concerns for the handlers. The reprogramming consists of two methods, non-integrating and integrating, as shown in Table 5. These depend on the efficiency, sustained expression and safety or clinical suitability.

Non-integrating reprogramming approaches, including Sendai virus, adenoviral vectors, episomal plasmids, minicircle DNA, EBV-derived episomes, synthetic mRNA, and direct protein transduction, have emerged as safer alternatives to integrating viral vectors. These methods avoid permanent insertion of reprogramming factors into the host genome, thereby producing “footprint-free” induced pluripotent stem cells (iPSCs) with markedly reduced risks of insertional mutagenesis, transgene reactivation, and oncogenic transformation [159,160]. This attribute makes them more suitable for clinical-grade iPSC production and aligns with regulatory expectations under current good manufacturing practice (cGMP) standards [157,161,162].

From a translational perspective, non-integrating approaches enhance the safety profile of iPSCs by minimizing genomic alterations, which is critical for therapeutic applications such as regenerative medicine, cell transplantation, and personalized disease modeling. However, these methods face significant technical and biological challenges. Compared to integrating vectors, they often exhibit lower reprogramming efficiencies, sometimes as low as 0.001% depending on the vector system [163], and typically require repeated delivery to maintain adequate factor expression. Moreover, transient expression may result in incomplete reprogramming, while certain systems such as adenoviral vectors or repeated mRNA transfection can introduce cytotoxic stress to the host cells [28]. Additionally, the manufacturing process for non-integrating reagents such as synthetic mRNA or minicircle DNA is more expensive and labor-intensive, further complicating large-scale or routine use [158].

Consequently, researchers often select reprogramming strategies based on their intended application. In basic research, where high reprogramming efficiency and stable transgene expression are prioritized, integrating vectors remain widely used despite their mutagenic risks. In contrast, non-integrating methods are preferentially employed in contexts where clinical safety and regulatory compliance are paramount, particularly in the generation of iPSCs intended for downstream therapeutic use. This strategic balance reflects a broader principle in stem cell biology; while efficiency and ease of use drive discovery at the bench, translational and clinical applications demand stringency in genomic integrity and long-term safety.

Another key consideration is that reprogramming somatic cells from endometriosis patients into induced pluripotent stem cells (iPSCs) requires coordinated, stepwise epigenetic remodeling to achieve a fully pluripotent state [164]. This process requires overcoming significant biological hurdles, most notably the mesenchymal-to-epithelial transition (MET), where mesenchymal stromal cells must robustly downregulate somatic markers like *Thy1* and *Snail* while upregulating E-cadherin-mediated cell–cell contacts to achieve pluripotency [165]. Many cells become “stalled” as pre-iPS intermediates, failing to activate core endogenous factors like *Oct4* and *Nanog* due to persistent promoter hypermethylation and a lack of global 3D nuclear reorganization. Furthermore, female-derived cells face the unique challenge of failing to reactivate the inactive X chromosome, which can distinguish human iPSCs from the “naive” pluripotency seen in embryonic stem cells and potentially affect X-linked gene expression relevant to the disease.

Despite these hurdles, recent advancements in 3D multicellular modeling are progressively bridging this gap. Key developments include significant improvements in iPSC reprogramming technologies, particularly the adoption of non-integrating methods that enhance genomic stability and clinical safety, refined and more reproducible lineage-specific differentiation protocols for generating endometrial epithelial and stromal cell types, and the emergence of organoid and assembloid systems that better recapitulate tissue architecture and cell–cell interactions. In parallel, advances in biomaterials and hydrogel-based scaffolds have enabled the reconstruction of physiologically relevant extracellular matrices, while microfluidic “organ-on-chip” platforms provide dynamic control over mechanical and biochemical cues [44]. Additionally, single-cell transcriptomics and other high-resolution omics approaches now allow precise characterization and validation of these models at cellular and molecular levels [166,167]. Collectively, these innovations have made iPSC-based 3D models increasingly feasible and powerful for studying complex diseases such as endometriosis.

## 7. Clinical Application and Future Perspective

The future perspective of endometriosis research using induced pluripotent stem cells (iPSCs) and organoid models holds great promise, but significant challenges remain in translating these technologies into clinical applications. Although Yang and Huang [168] highlighted the potential for clinical translation, they also pointed out critical gaps, such as safety concerns with hiPSCs, the need for optimal reprogramming methods, and the necessity of clinical trials to validate efficacy and safety. Building on these concerns, it becomes clear that technological advancement alone is not sufficient; rigorous validation and standardization must accompany innovation to ensure reliable translational outcomes. Additionally, Brassard and others [169,170] emphasized the importance of incorporating vascularization into organoids to improve their size, complexity, and physiological relevance, as current models are limited by oxygen and nutrient diffusion constraints. Bloise et al. [170] noted that focusing primarily on stromal and epithelial cells is insufficient, advocating for the inclusion of diverse cell types, such as immune and vascular cells, to create comprehensive models that better reflect the endometrium’s complexity. These insights collectively highlight a growing recognition that future models must evolve toward greater biological fidelity if they are to capture the dynamic interplay of cells and signals that drive endometriosis. Concerns about long-term efficacy and tumor formation, as mentioned by [171], also need to be addressed to ensure the safety of these therapies. Furthermore, while Yang and Huang [168] also discussed the potential for personalized medicine, more research is required to develop specific strategies and examples that tailor treatments to individual patients based on their genetic makeup and disease characteristics. Advancing this field will therefore demand not only improved models, but also deeper integration of molecular data, clinical phenotyping, and patient-specific disease signatures.

Parallel to these scientific challenges, the field must address the logistical and economic realities that often determine whether a technology moves beyond the laboratory. A primary barrier is the high cost per patient line, with research-grade iPSC generation estimated between USD 10,000 and USD 25,000, while clinical-grade lines can reach USD 800,000 [172]. Additionally, the time to validation is substantial, typically requiring 6 to 9 months from patient recruitment to full characterization [172]. These constraints are further complicated by significant clone-to-clone variability, which can lead to inconsistent phenotypes even within lines derived from the same genetic background [40]. These issues are compounded by a lack of standardized endometrial differentiation protocols, which are often characterized by low reproducibility and yields [173].

To conclude, endometriosis is a highly heterogeneous disease, with significant variation in genetic, epigenetic, hormonal, and immunological profiles among patients. To capture this complexity and uncover disease mechanisms or potential biomarkers, it is essential to establish comprehensive iPSC biobanks derived from a diverse range of patient samples. By creating large-scale collections of endometriosis-derived iPSCs, researchers can perform comparative studies that account for differences in disease stage, lesion type, anatomical location, and individual genetic predispositions [172]. Such resources would not only accelerate the discovery of molecular subtypes but also create a robust platform for testing personalized therapeutic strategies. Ultimately, this resource would lay the foundation for more precise and individualized strategies in understanding and managing endometriosis.

## Figures and Tables

**Table 1:** Representative Genomic Loci and Candidate Risk Genes.

Locus	Candidate Gene(s)	Biological Implication	Reference
6q25.1	*ESR1*	Estrogen receptor signaling	[60]
11p14.1	*FSHB*	Hormone metabolism	[60]
6p25.2	*SYNE1*	Nuclear envelope integrity	[60]
7p15.2	*rs12700667*	Risk for moderate-severe disease	[62]
2q35	*FN1*	Cell adhesion and migration	[60]

**Table 3:** Summaries of recent insights into disease progression obtained from studies using the disease model.

Category	Evidence
Hormone Responsiveness and Gland-like Structures	Organoids recapitulate glandular architecture; glycogen production Periodic Acid-Schiff (PAS+) and MUC-1 luminal secretion [130]. Established stable, hormone-responsive endometrial organoids [21,22].
Disease-Specific Traits	Patient-derived organoids (PDOs) retain key molecular features Mismatch repair (MMR), tumor protein 53 (p53), Polymerase Epsilon (POLE) mutations [131]. High concordance in morphology and genetic alterations with patient tumors [132]. Captured clinical heterogeneity in endometriosis and cancer organoids [133].
Long-term Expansion and Stability	Maintained chromosomal stability (46,XX); Endometrial Extracellular Matrix (EndoECM) supports expansion [130]. Long-term expansion of the cultures > 6 months while maintaining the molecular and histological characteristics of the original glandular epithelium [21,22]. ~71% success rate for long-term organoid establishment [131].
Drug Testing	PDOs show differential drug responses compared to 2D models [131]. Suitable for high-throughput drug screening [132].
Signaling Pathway Studies	(Trastuzumab Deruxtecan) T-Dxd reduces and increases phosphorylated Extracellular Signal-Regulated Kinase 1 and 2 (pERK1/2) signaling [132]. Maintenance depends on WNT activation and Transforming Growth Factor-beta and Bone Morphogenetic Protein (TGFβ/BMP) inhibition [130].
Modeling Early Disease Events	Enables study of ECM remodeling in early lesion formation [130]. Ascites-derived organoids model metastasis and advanced disease [131].
Functional Validation	Confirmed epithelial identity (CK+/vimentin−) and stemness markers [130]. Organoids resemble original tissue morphology and structure [131].
Therapeutic Testing	Combination therapy (T-Dxd + MEK inhibitors) reduces growth [132]. Validated responses to paclitaxel and carboplatin [131].

**Table 5:** Comparison of Integrating vs. Non-Integrating Reprogramming Methods.

Feature	Integrating Methods	Non-Integrating Methods	Ref.
Genome Integration	Permanent integration of transgenes into host genome	No genomic integration	[155]
Safety profile	Higher risk of insertional mutagenesis, transgene reactivation, oncogenic transformation	Lower mutagenic risk is preferred for clinical-grade iPSC production	[156]
Reprogramming Efficiency	Generally high; stable long-term expression of factors	Lower efficiency; may require repeated delivery to sustain expression	[157]
Expression stability	Sustained, stable transgene expression	Transient expression; risk of incomplete reprogramming	[28]
Technical and Cost	Relatively easy to use; widely available kits and protocols	Higher complexity; mRNA/minicircle production is labor-intensive and costly	[158]
Primary Usage	Ideal for basic research requiring high efficiency and stable expression	Clinical- or translational-grade iPSC generation	[159]

## Data Availability

No new data were created or analyzed in this study. Data sharing is not applicable to this article.
